# A Case in Which the Application of a Small Quantity of Strychina Was Followed by Unpleasant Effects

**Published:** 1844-04

**Authors:** W. L. Sutton

**Affiliations:** Georgetown, Ky.


Art. II.—*A Case in which the application of a small quantity of Strychnia was followed by unpleasant effects.* By W. L. Sutton, M.D., of Georgetown, Ky.

Mr. Davis, apothecary of this place, in weighing strychnine, dropped a little on his finger which, at the time, happened to be moist. He wiped it off carelessly, but in the course of an hour his hand and arm began to swell and feel numb. The swelling of the hand and fore arm was very considerable, attended by loss of sensation, (but not of motion), and severe pain in the shoulder. He used sinapisms and free friction with ammoniated liniment to the arm, and took morphia and spirits of ammonia internally. Next morning the swelling was gone, but he continued to feel slight shooting pains in his hand for some days, and even yet, he feels some occasionally. A depression took place in the bulb of the finger, and still continues. It has very much the appearance, as if produced by a condensation of the cellular substance beneath the skin. On two previous occasions he had felt slight pains from dropping some of the same article on his hand. He knew a similar effect in one of his friends. I will add, that I have heard that a man in Fayette county, whilst preparing some strychnine to poison crows, dropped some on an ulcer upon his hand, which caused his death in a few hours. I can say nothing as to the truth of this report. It is evident that certain persons have a peculiar susceptibility to its influence, as Mr. Davis thinks not more than the fifteenth part of a grain could have fallen upon his finger. This may suggest a caution in applying this article to denuded surfaces, seeing that so minute a quantity, upon the thick skin on the bulb of the finger, may be followed by distressing effects.

February, 1844.

